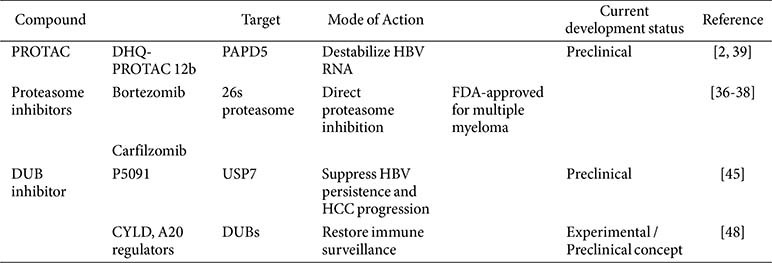# The Ubiquitin-Proteasome System in Hepatitis B Virus Infection and Hepatocarcinogenesis: Viral Manipulation and Therapeutic Targets

**DOI:** 10.4014/jmb.2508.08005

**Published:** 2025-10-27

**Authors:** Jiwoo Han, Kyun-Hwan Kim, Sang-Uk Seo

**Affiliations:** 1 Department of Microbiology, College of Medicine, The Catholic University of Korea, Seoul 06591, Republic of Korea; 2 Department of Precision Medicine, School of Medicine, Sungkyunkwan University, Suwon 16419, Republic of Korea; 3 Department of Medical Sciences, Graduate School, The Catholic University of Korea, Seoul 06591, Republic of Korea

**Keywords:** Hepatitis B virus, ubiquitin-proteasome system, deubiquitination, hepatocellular carcinoma, proteasome inhibitors, PROTACs

## Abstract

Hepatitis B virus (HBV) infection is a major cause of hepatocellular carcinoma. To sustain viral life cycle, HBV modulates host pathways controlling protein turnover and innate immunity. Critical events in the HBV life cycle include the conversion of relaxed-circular DNA to covalently closed circular DNA, transcriptional activation of viral genes, and the synthesis and maturation of structural and regulatory proteins. These processes are tightly linked to the ubiquitin-proteasome system (UPS), the central machinery for post-translational control. Disruption of UPS homeostasis impairs antiviral signaling and drives malignant progression. Among HBV proteins, HBV X protein reshapes protein ubiquitination by recruiting or redirecting host E3 ligases. These alterations elevate the stability of virus-facilitating mediators, downregulate interferon-stimulated responses, and expedite the turnover of key tumor suppressors. In parallel, the host counters by attaching degradative ubiquitin chains to viral antigens or downregulating proviral modifications through deubiquitinases (DUBs). In this review, we consolidate current knowledge of the HBV-UPS interplay, dissecting molecular circuits that govern ubiquitin-driven degradation and detailing the specific E3 ligases hijacked by the virus. Finally, we evaluate therapeutic potentials that target HBV-UPS interaction, ranging from broad-spectrum proteasome inhibitors and selective DUB antagonists to proteolysis targeting chimeras.

## Introduction

Hepatocellular carcinoma (HCC) remains one of the most lethal malignancies worldwide, and chronic hepatitis B virus (HBV) infection is responsible for a large fraction of these cases [1-3]. The compact, partially double-stranded viral genome of 3.2 kb encodes four overlapping open reading frames, S, P, C, and X, that collectively direct assembly, replication, and host modulation [4]. Among these, the HBV X protein (HBx) performs multiple regulatory functions that influence viral replication, host interactions, and disease progression [5]. HBx is implicated in regulating cellular signaling pathways, cell growth, gene transcription, and apoptosis during HCC progression [6, 7]. HBx also acts as a positive regulator of virus replication by stimulating four viral promoters [8-11]. While HBx clearly shapes HBV replication, the underlying mechanisms of its interplay with the host defense system remain poorly understood, highlighting the need for further investigation.

During the past decade, numerous studies have detailed how HBV manipulates the ubiquitin-proteasome system (UPS). This proteolytic pathway is composed of E1 activating enzymes, E2 conjugating enzymes, and E3 ligases. These enzymes attach polyubiquitin chains that guide substrates toward degradation by the 26S proteasome [3, 12]. Deubiquitinating (DUB) enzymes further modulate proteostasis by editing or removing ubiquitin moieties [13].

Under normal conditions, UPS-mediated protein turnover restricts viral infection by removing viral proteins and sustains innate immunity. HBV bypasses these systems by engaging cellular ligase complexes. For example, HBx binds E6-associated protein (E6AP) [14] and the Cullin4-RING (CRL4-RING) ligase assembled around DNA damage-binding protein 1 (DDB1) [15]. Through these interactions, ligase activity is redirected to stabilize viral factors, suppress interferon signaling, and accelerate degradation of tumor suppressors such as p53. The effect promotes immune evasion and establishes a cellular milieu receptive to malignant transformation.

Comparable exploitation of the UPS has been documented not only in HBV but also in other human viruses. Human papillomavirus E6 recruits E6AP to target p53 [27]. Human immunodeficiency virus-1 variance inflation factor (Vif) forms a complex with cullin (CUL) to ubiquitinate and degrade apolipoprotein B mRNA editing enzyme catalytic polypeptide-like 3G (APOBEC3G) [28]. Influenza A virus reduces innate sensing by directing tripartite motif-containing protein 25 (TRIM25) for degradation [21, 29]. Severe acute respiratory syndrome coronavirus 2 encodes an open reading frame that interacts with CUL2-based ligases [30]. These parallels highlight the evolutionary advantage conferred by commandeering host ubiquitination machinery.

The present review integrates current knowledge of HBV-mediated UPS remodeling, focusing on ubiquitin-dependent circuits controlled by HBx and other viral components. It also surveys host-directed interventions, including proteasome inhibitors, selective DUB modulators, and Proteolysis-targeting chimeras (PROTACs), that aim to disrupt viral persistence and mitigate virus-induced carcinogenesis.

### Molecular Interplay between HBV and the UPS

HBx redirects host E3 ligase activity by mimicking DDB1 and CUL4-associated factor 1 (DCAF) adaptors to bind DDB1. This interaction repurposes the CRL4-DDB1 complex toward chromosomes 5/6 complex (Smc5/6), whose proteasomal removal represses covalently closed circular DNA (cccDNA) transcription, a mechanism confirmed by clustered regularly interspaced short palindromic repeats (CRISPR) screens and structural studies [16-18]. Recruitment of E6AP by HBx similarly drives p53 ubiquitination and degradation, blunting apoptosis under genotoxic stress and promoting hepatocyte survival [14]. In addition, interference with β-catenin turnover sustains wingless-related integration site (Wnt) signaling and enhances proliferation linked to oncogenesis [12].

Beyond E3 ligase hijacking, HBV reshapes DUB activity, such as upregulation of ubiquitin-specific protease 7 (USP7). This upregulation stabilizes MDM2 and accelerates p53 loss in a self-reinforcing cycle favoring viral persistence [19]. In addition, downregulation of cylindromatosis (CYLD) and A20 impairs nuclear factor kappa B (NF-κB) control and skews immunity toward tolerance [3]. Ubiquitination of HBV core (HBc) triggers capsid disassembly and genome import [20]. Additionally, HBV large surface antigen (LHBs) is routed into endoplasmic reticulum (ER)-associated degradation, which induces chronic ER stress and drives hepatocyte damage and neoplastic progression [21].

Collectively, UPS alterations coordinate cccDNA maintenance, chronic infection, immune evasion, and oncogenic signaling. Therefore, mapping these virus-host interfaces could uncover UPS components as strategic targets for next-generation antiviral and anticancer therapies.

### UPS-Mediated Regulation of Host Antiviral Immunity and Oncogenic Signaling

Modulation of the UPS by HBV exerts profound effects on both innate immunity and pathways linked to oncogenic transformation. HBx suppresses the RIG-I/MAVS-TBK1-IRF3 pathway by recruiting host DUBs. These DUBs accelerate mitochondrial antiviral-signaling protein (MAVS) degradation and remodel K63-linked ubiquitin chains, thereby collectively diminishing type I interferon output [22]. Concurrent loss of tumor necrosis factor receptor associated factor 3 (TRAF3) and TRAF6 which are E3 ligases essential for NF-κB activation and cytokine expression, further impairs antiviral responses [14]. At the same time, proteasomal removal of suppressor of cytokine signaling 3 (SOCS3) sustains signal transducer and activator of transcription 3 (STAT3) phosphorylation. This prolonged STAT3 phosphorylation is a common feature of HBV-associated HCC and is closely linked to inflammation-driven carcinogenesis [23].

Tumor suppressors and cell-cycle regulators are also influenced by UPS during chronic infection. Proteasome-dependent depletion of p53 compromises DNA damage surveillance and apoptotic responses [14]. Accelerated turnover of cyclin-dependent kinase (CDK) inhibitors p21 and p27 induces unchecked proliferation and accumulation of genomic lesions [24, 25]. Furthermore, interference with ubiquitin tagging or disruption of β-TrCP ligase activity stabilizes β-catenin and activates transcriptional programs that support hepatocyte survival and proliferation [26, 27].

By reshaping ubiquitination and DUB processes, HBV creates an intracellular environment that both downregulates immune surveillance and reprograms signaling networks toward malignant progression. A detailed molecular understanding of these UPS alterations promises to reveal novel targets for interventions aimed at disrupting viral persistence and preventing HCC. These immune-evasive and growth-promoting changes accumulate over the course of chronic infection, laying a molecular foundation for malignant conversion.

### UPS Dysregulation in HBV-Associated HCC

Persistent HBV infection remains a major cause of HCC and increasing data point to long-term disruption of the UPS as a catalyst for this malignant shift [28]. Continuous remodeling of protein-turnover pathways strengthens viral maintenance. Simultaneously, this reconfigures cellular homeostasis in ways that favor oncogenesis. A prominent route to malignant transformation involves proteasome-directed elimination of critical genome guardians. Tumor suppressors such as p53 [14], Smc5/6 [29], and UBX domain-containing protein 7 (UBXN7)[30] undergo accelerated degradation after recruitment of host E3 ubiquitin ligases by the HBx. Two ligase platforms, such as DDB1-CUL4 and seven-in-absentia homologue 1 (Siah-1), feature prominently in this process and change the cellular balance toward unchecked proliferation and genomic instability [15, 31]. UPS dysregulation also amplifies inflammatory and fibrogenic pathways related to HBV-related liver disease. Stabilization of β-catenin or removal of IκBα propagates oncogenic circuits governed by Wnt/β-catenin, NF-κB, and STAT3 signaling, all frequently deregulated in HBV-related HCC [13, 32]. The sustained activity of these pathways under chronic infection links persistent inflammation to tumor development.

Altered expression or activity of UPS components may provide clinically useful biomarkers or therapeutic insights. Loss of Smc5/6 or UBXN7 [29] and atypical patterns of specific ligases or DUBs [34] correlate with immune suppression and cancer progression. Conversely, host proteins can also optimize the environment for viral replication and HCC development by inhibiting the proteasomal degradation of HBx, which is essential for viral replication and acts as a viral oncoproteins. The cellular FADD-like IL-1β-converting enzyme (FLICE)-like inhibitory protein (c-FLIP) is a key factor that inhibits apoptosis. Our previous reports showed that HBx is stabilized by c-FLIP_L_ or c-FLIP_S_, thereby protecting it from proteasome-mediated degradation and contributes to robust HBV transcription [33].

**Fig. 1:**
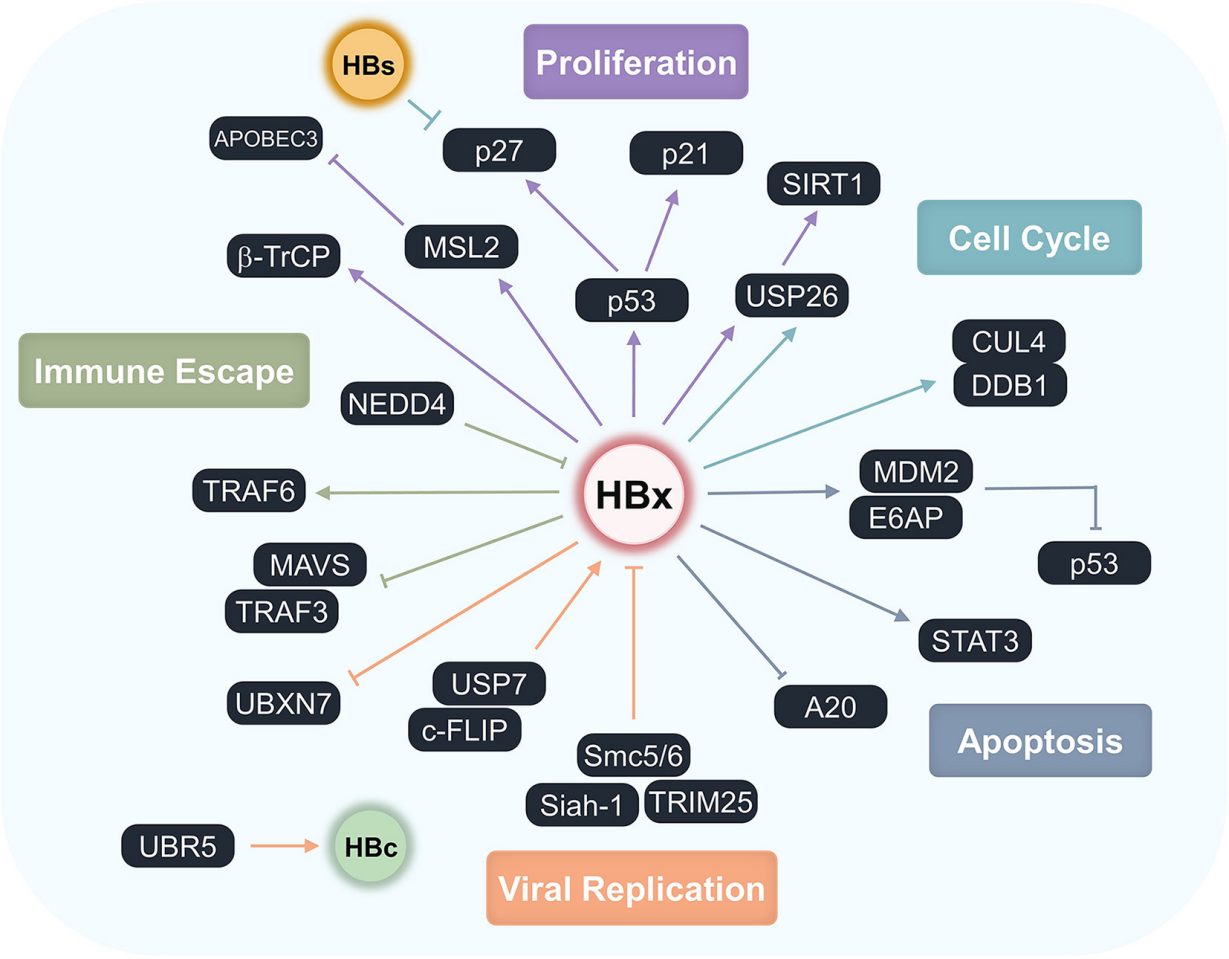
HBV-related modulation of the UPS and downstream cellular pathways. HBV manipulates host UPS by regulating host E3 ligases and DUBs, thereby modifying the stability of viral and host proteins. HBV recruits or interferes with multiple UPS components, including E6AP, MDM2, USP7, USP26, CUL4-DDB1, NEDD4, TRAF3/6, TRIM25, and Siah-1, as well as host restriction factors such as Smc5/6 and APOBEC3B. These interactions influence apoptosis, proliferation, cell cycle progression, immune escape, and viral replication. The schematic highlights the dynamic interactions between UPS-related host proteins and HBV proteins, particularly HBx. The color of each line corresponds to the cellular effect indicated that color.

Collectively, these strategies show how HBV regulates the UPS to survive within the host and sustain viral infection. Representative interactions are summarized in Table 1, while Fig. 1 provides a mechanistic overview of UPS-related oncogenic pathways. Together, these complementary illustrations show that HBV–UPS crosstalk affects multiple host proteins to drive immune escape, cell proliferation, apoptosis, cell cycle dysregulation, inflammation, and viral replication. Incorporating UPS-related signatures into diagnostic workflows may therefore improve early detection, prognostication, and therapeutic planning for HBV-associated HCC.

### UPS Modulation as an Antiviral Strategy for HBV

Recognizing its central role in viral persistence, immune suppression, and tumorigenesis [34, 35], therapeutic efforts have shifted toward modulating UPS function rather than directly targeting viral enzymes. Bortezomib and carfilzomib, first approved for multiple myeloma, preserve tumor-suppressive factors and reduce viral protein levels by inhibiting proteasomal activity in HBV models. However, their clinical utility in liver disease is disrupted by off-target toxicity and limited specificity [36-38]. Given the clinical utility of proteasome inhibitors such as bortezomib and carfilzomib, further investigation might be useful to determine whether other proteasome inhibitors can also exert effective anti-HBV activity.

To overcome several drawbacks of proteasome inhibitors, recent research has advanced more precise degradation technologies. PROTACs couple target proteins to E3 ligases, prompting ubiquitination and subsequent proteasomal destruction [2, 39]. For instance, Li *et al*. reported dihydroquinolizinone-based PROTACs that restrain the non-canonical poly(A) polymerase PAP-associated domain-containing protein 5 (PAPD5) to the cereblon ligase. This led to PAPD5 ubiquitination and degradation at low concentrations. These compounds also suppressed both HBV and hepatitis A virus replication in HepG2-NTCP cells with minimal cytotoxicity [39]. Therefore, applying similar PROTACs against HBx-regulated host factors could achieve concurrent inhibition of viral replication and oncogenic signaling while avoiding the systemic toxicity of first-generation proteasome inhibitors [40].

On the other hand, there is also an attempt to develop antiviral drugs using small chemicals that promote the proteasome-dependent degradation of HBx. Dicumarol, an inhibitor of NAD(P)H:quinone oxidoreductase 1 (NQO1), reduced cccDNA transcription by promoting degradation of HBx, an essential activator of cccDNA transcription, and ultimately showed potent antiviral activity [41].

Several studies also seek to inhibit DUBs during virus infection. Blocking enzymes such as ovarian tumor domain-containing protein 5 [42] or USP26 [43] can promote the removal of HBV-stabilized oncoproteins, reactivate antiviral signaling, and enhance apoptosis in HCC cells. Because certain DUBs regulate immune checkpoint molecules, including PD-L1 [44], their inhibition could complement immune-based therapies. Another study showed that HBV manipulates the host's USP7 to create an environment favorable for viral replication [45]. Inhibiting USP7 can stabilize tumor suppressor proteins like p53 and enhance the antiviral efficacy of existing drugs like entecavir, highlighting DUB inhibition as a promising new therapeutic strategy for HBV infection.

Table 2 summarizes representative compounds that target the UPS, their mechanisms of action, and current stages of development, ranging from preclinical studies to FDA-approved clinical applications [2, 3, 36-39, 45].

Finally, combination regimens are also under investigation, pairing UPS-directed agents with nucleos(t)ide analogues or immunotherapies [46, 47]. Such approaches aim to limit viral reservoirs while strengthening host immunity against infected or transformed hepatocytes, thereby offering a multifaceted strategy for HBV infection.

## Conclusions and Perspectives

Persistent HBV infection ranks among the foremost contributors to global HCC burden [3, 19], and UPS dysregulation has been implicated in sustaining viral replication while driving the malignant shift characteristic of chronic disease [26, 28, 29]. By securing both ubiquitination and DUB machinery, immune clearance is evaded, antiviral signaling pathways are downregulated, and tumor suppressors are rapidly degraded, thereby establishing a microenvironment permissive to oncogenesis [14, 16]. As a result, therapeutic efforts have focused on strategies targeting the HBV-UPS axis. Representative approaches include proteasome inhibitors, PROTACs, and DUB antagonists, which have demonstrated the capacity to lower viral burden, reinstate host immunity, and interrupt tumor-promoting signaling [2, 28, 39, 42].

**Fig. 2:**
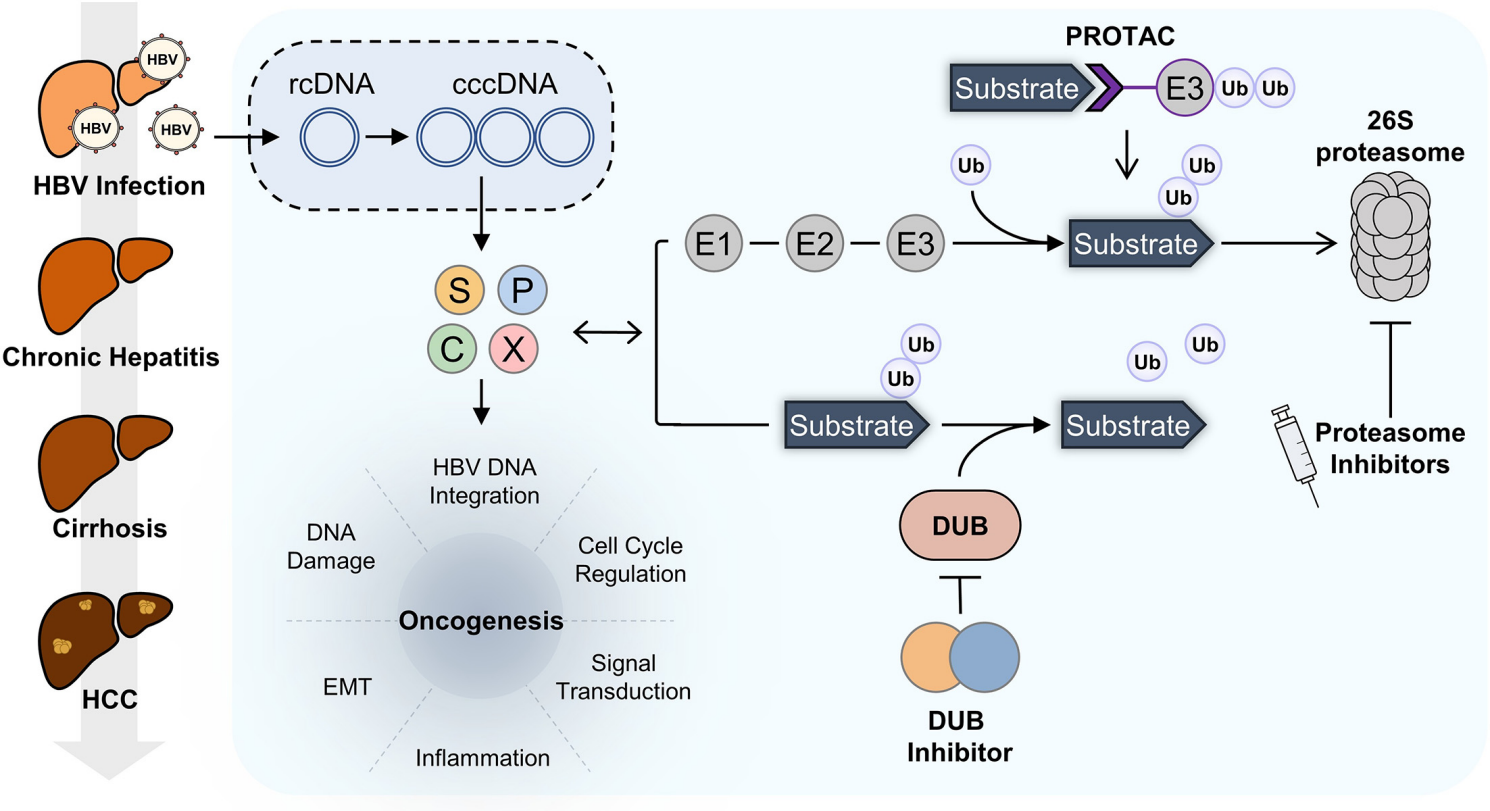
Host UPS and possible therapeutic strategies in HBV-infected cells. Upon infection, rcDNA is delivered into the nucleus and converted into cccDNA using host enzymes. HBV cccDNA serves as the template for surface (S), polymerase (P), core (C), and HBx (X) proteins. HBV proteins regulate numerous cellular processes such as inflammation, HBV DNA integration, DNA damage, cell cycle regulation, epithelial-mesenchymal transition (EMT), and signaling pathways. HBV proteins are also involved in modulating host UPS to sustain viral infection. Ubiquitination is mediated by a sequential enzymatic cascade involving E1 activating enzymes, E2 conjugating enzymes, and E3 ligases, which collectively attach ubiquitin to target substrates. The ubiquitinated substrates are subsequently recognized and degraded by the 26S proteasome. In contrast, DUBs counteract this process by removing ubiquitin from substrates, thereby regulating protein stability. Therapeutic strategies have been developed to modulate this process such as proteasome inhibitors, which block the 26S proteasome. PROTACs redirect specific proteins for ubiquitination and degradation by recruiting E3 ligases. DUB inhibitors prevent DUB-mediated removal of ubiquitin chains. These approaches could restore antiviral immune responses and attenuate oncogenic progression.

Fig. 2 provides a comprehensive overview of HBV-induced liver disease progression and its interplay with the UPS as well as representative therapeutic strategies described above. In conclusion, targeting UPS can serve as a strategic intervention point in HBV-associated liver disease, and detailed mapping of viral exploitation of UPS pathways is expected to improve pathogenetic models and accelerate next-generation antiviral and anticancer drug development.

## Figures and Tables

**Table 1:** UPS-mediated regulation of viral proteins during HBV infection.

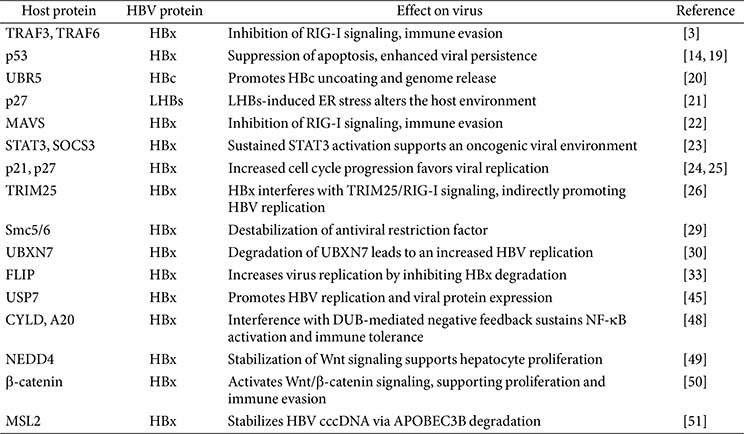

**Table 2:** Lists of compounds targeting the UPS and their therapeutic applications.